# Empathy, Yes, and…, Improving Dementia Care Through Improv

**DOI:** 10.1093/geroni/igaf122.289

**Published:** 2025-12-31

**Authors:** Candace Kemp, Jennifer Morgan, Christina Cifuentes, Mary Jones

**Affiliations:** Georgia State University, Atlanta, Georgia, United States; Georgia State University, Atlanta, Georgia, United States; Georgia State University, Atlanta, Georgia, United States; Georgia State University, Atlanta, Georgia, United States

## Abstract

Empathy is at the heart of person-centered dementia care and has a significant impact on care interactions and relationships. Some family caregivers of persons living with dementia instinctively approach care with empathy. Yet, many have difficulty relating to their person, particularly when faced with behaviors perceived as out-of-character, unexpected, or challenging. Identifying ways to enhance caregiver empathy is critical to improving dementia care experiences. One innovative and promising avenue involves improvisational (i.e., “improv”) theatre. Improv, like empathy, requires actively listening and observing, collaborating, meeting people where they are, and being in the moment, and can enhance caregivers’ engagement capacity. In this paper we examine improv’s potential by analyzing data collected as part of a pilot study testing, “Improving Care through Improv,” an 8-hour training program designed for family and friends caring for someone with moderate dementia. We assessed the program through a no-control study involving surveys (collected at baseline, program completion, and 3-months post-baseline) and focus groups (n = 4). Forty caregivers participated in the training and completed the study. Training was associated with decreases in stress, negative responses to challenging behaviors, and reports of frustration. In open-ended survey and focus group data, most referred to a “mindset shift,” including many who described enhanced capacity to be “more patient,” “accept and understand,” have “more grace,” and “not always “correct.” One explained, understanding the “need to radically accept where my person is” and “support her from there.” This training demonstrates improv’s ability to impact empathy and care relationships and requires additional systematic investigation.

